# MALDI-TOF analysis of blood serum proteome can predict the presence of monoclonal gammopathy of undetermined significance

**DOI:** 10.1371/journal.pone.0201793

**Published:** 2018-08-02

**Authors:** Francisca Barceló, Rosa Gomila, Ivan de Paul, Xavier Gili, Jaume Segura, Albert Pérez-Montaña, Teresa Jimenez-Marco, Antonia Sampol, José Portugal

**Affiliations:** 1 Grupo de Investigación Clínica y Traslacional, Departamento de Biología Fundamental y Ciencias de la Salud, Instituto Universitario de Investigación en Ciencias de la Salud (IUNICS), Universitat de les Illes Balears, Palma de Mallorca, Spain; 2 Instituto de Investigación Sanitaria Illes Balears (IdISBa), Palma de Mallorca, Spain; 3 Servicios Cientificotécnicos, Universitat de les Illes Balears, Palma de Mallorca, Spain; 4 Grupo de Sistemas Electrónicos, Universitat de les Illes Balears (GSE-UIB), Palma de Mallorca, Spain; 5 Servicio de Hematología y Hemoterapia, Hospital Universitario Son Espases, Palma de Mallorca, Spain; 6 Fundació Banc de Sang i Teixits de les Illes Balears, Gobierno Balear, Palma de Mallorca, Spain; 7 Instituto de Diagnóstico Ambiental y Estudios del Agua, CSIC, Barcelona, Spain; Universitat des Saarlandes, GERMANY

## Abstract

Monoclonal gammopathy of undetermined significance (MGUS) is a plasma cell dyscrasia that can progress to malignant multiple myeloma (MM). Specific molecular biomarkers to classify the MGUS status and discriminate the initial asymptomatic phase of MM have not been identified. We examined the serum peptidome profile of MGUS patients and healthy volunteers using MALDI-TOF mass spectrometry and developed a predictive model for classifying serum samples. The predictive model was built using a support vector machine (SVM) supervised learning method tuned by applying a 20-fold cross-validation scheme. Predicting class labels in a blinded test set containing randomly selected MGUS and healthy control serum samples validated the model. The generalization performance of the predictive model was evaluated by a double cross-validation method that showed 88% average model accuracy, 89% average sensitivity and 86% average specificity. Our model, which classifies unknown serum samples as belonging to either MGUS patients or healthy individuals, can be applied to clinical diagnosis.

## Introduction

Monoclonal gammopathy of undetermined significance (MGUS) is a pathological condition in which plasma cells undergo a proliferative disorder associated with a lifelong risk of progression to malignant multiple myeloma (MM) [1,2]. MGUS is defined as having serum M-protein (monoclonal immunoglobulin) < 3 g/dL, less than 10% clonal plasma cell population in the bone marrow, and the absence of end-organ damage [3,4]. In people aged 50 years and older, MGUS is the most common plasma cell dyscrasia [5]. At present, a lifelong clinical follow-up of individuals diagnosed as having MGUS is suggested because patients often remain stable for years without treatment. Although little is known about the events that promote the evolution of MGUS and its progression to MM [6–8], patients who develop MM have been, in most cases, previously diagnosed with MGUS [1,2,7,9]. Therefore, the diagnosis and the clinical control of MGUS status are important for an earlier detection and treatment of MM and its better clinical performance [10]. While diagnostic to detect the transition of MGUS to MM at an early stage is based on repeated blood tests, X-rays analysis, and bone marrow biopsies, it seems necessary to develop alternative methods to characterize the MGUS disease status. Several boundaries exist in the capacity of laboratory assays for detecting and discriminating between the MGUS status and the initial asymptomatic phase of MM, thus a variety of tests are used to encompass the diverse nature of the M-protein [11,12]. Hence, an enhanced interest exists in developing new analytical approaches such as differential scanning calorimetry of diseased blood serum and to improve the new MALDI-TOF mass spectrometry methods [12–14].

Proteomic techniques are useful to describe novel biomarkers in diseased serum because differences in serum protein and peptide profiles can provide potential insights into the MGUS status and its transition to MM, consistent with the changes observed in gene expression [15,16]. In this context, both MGUS and MM show peculiarities in the blood serum proteome, which have been examined by differential scanning calorimetry (DSC), in which thermogram parameters can distinguish patients having MGUS or MM from healthy people [13,14,17,18]. Further work on biomarkers for MGUS in biological fluids is required to establish DSC and changes in blood proteome as reliable diagnostic tools, and for distinguishing the disease status in MGUS patients.

MALDI-TOF mass spectrometry is a powerful tool for sample differentiation and identification of proteomic markers in biofluids [19–23]. It can complement the diagnostic equipment available for clinical diagnosis [12,19,23], and it has been used for the proteomic analysis of MM [24,25]. MGUS patients would contain certain proteins described as having peculiar patterns of expression in this dyscrasia [26]. Proteomic profiling of serum samples has detected markers that would be indicative of early event pathogenesis and disease progression in MGUS patients [27].

Here, we used MALDI-TOF to examine sera obtained from MGUS patients and a control group of healthy volunteers. The main objective of our approach was to build and validate a predictive model to be used for the clinical diagnosis of individuals suffering from MGUS. The goal was to achieve the highest prediction quality without identifying individual proteins as putative biomarkers. The predictive model was built using a support vector machine (SVM) supervised learning method tuned by applying a 20-fold cross-validation scheme. The generalization performance of our predictive model was evaluated by a double cross-validation method.

## Materials and methods

### Materials

Trifluoroacetic acid (TFA) and acetonitrile (CH_3_CN) were from Sigma-Aldrich (Madrid, Spain), and alpha-cyano-4-hydroxycinnamic acid (CHCA) from Fluka Analytical (Sigma-Aldrich, Madrid, Spain). Reversed-Phase C18 Resin ZipTip Pipette Tips were purchased from Millipore (Madrid, Spain).

### Study population and institutional approval

A total of 103 patients clinically diagnosed with MGUS at the University Hospital Son Espases (HUSE) (Palma de Mallorca, Spain), as well as 108 healthy volunteer donors (HC) from the “Fundació Banc de Sang i Teixits de les Illes Balears” (Gobierno Balear, Spain) were recruited for this study. Demographic details and clinical characteristics are displayed in Table 1. The Clinical Research Ethics Committee of the Balearic Islands (CEIC-IB) approved both the study protocol and patient consent procedures (IRB#: IB 1914/12 MB). All the enrolled volunteers gave written informed consent for their blood to be used in this study. Serum collection and processing were done according to the analytical protocols of the Hospital. Samples were aliquot and de-identified by the Biobank HUSE (University Hospital Son Espases) or the “Fundació Banc de Sang i Teixits de les Illes Balears” (Gobierno Balear, Spain) and stored at -80°C until they were delivered to the basic science research team. In this way, all samples were anonymized and blinded for unbiased data collection as described previously [13]. The associated demographic information was collected by the clinical study personnel and provided to the basic science team for data analysis (Table 1).

**Table 1:** Patient demographics and disease characteristics.

Sample set^a^	Number of samples	Male/Female	Age range	Age (Mean ± SD)	M-protein (g/dL) (Mean ± SD)
Healthy controls	108	67/41	21–66	46 ± 9	Below cut off
MGUS patients^b^	103	50/53	41–88	66 ± 12	0.65±0.41

^a^ All serum samples were from Caucasian people. Clinical data were collected at the time of diagnosis.

^b^ MGUS encompasses serum samples of the following isotypes: IgG κ (38), IgA κ (12), IgM κ (7), IgG λ (27), IgA λ (13), IgM λ (2), IgG κ + IgM κ (1), IgG κ + IgM λ (1), IgA κ + IgA λ (1), IgM κ + IgM λ (1).

The diagnosis of MGUS was based on standard clinical criteria [28]. Serum samples were classified according to the monoclonal serum protein as: IgG κ, IgG λ, IgA κ, IgA λ, IgM κ, and IgM λ. MGUS patients had serum M-protein concentration < 3 g/dL. The control group consisted of healthy apheresis blood donors (HC). The presence of monoclonal protein was ruled out by performing total protein and serum protein electrophoresis testing in all samples from apheresis blood donors at the first donation and at least every year thereafter, according to the National and European regulations (available at: https://www.edqm.eu/en/blood-transfusion-guides-1608.html). All HC serum samples were negative in analytical tests for HIV, Hepatitis B and C, and *Treponema pallidum* infections.

### Serum sample collection and preparation

Serum samples of MGUS patients were obtained at the time of routine clinical procurement. Samples from healthy apheresis blood donors were obtained of volunteer donation. Sample collection and handling were conducted according to the approved experimental protocols of the hospital, as described elsewhere [13]. In brief, blood was collected in 9 mL red-top glass tubes with serum clot activator (Vacuette España, San Sebastian de los Reyes, Spain), allowed to sediment for 30 min at room temperature and centrifuged at 4000 rpm in a Heraeus Megafuge (Heraeus, Madrid, Spain) for 15 min. Pooled samples were aliquot and immediately stored frozen at -80°C until their use within one month. No aliquot underwent more than one freeze-thaw cycle before analysis. Collected serum samples were randomly divided into two groups: a training set (95 MGUS and 100 healthy controls (HC) serum samples) and a blinded test set (consisting of 8 MGUS and 8 HC serum samples).

Serum samples were purified and concentrated by using reversed phase C18 Resin ZipTip pipettes, following the manufacturer’s instructions. 12 μl serum was mixed with 3 μl of 5% TFA and applied to the C18-ZipTip Pipette. The solution was passed through the Zip-Tip pipette repetitively (20 times). After washing with 10 μl of 0.1% TFA, the bound proteins/peptides were eluted with 6 μl of 0.1% TFA:CH_3_CN (1:2, v/v). The eluted proteome fraction was mixed with 6 μl of CHCA matrix solution (5 mg of CHCA in 1 ml of 0.1% TFA:CH_3_CN, 1:3, v/v) and 2 μl of this mixture were spotted on the MTP 384 target plat polished steel (Bruker Daltonics, Leipzig, Germany) and air-dried. Serum samples were randomly selected and measured in experiments conducted in the same and different days. Three-six technical replicate spectra were obtained for each sample. Biological replicates of MGUS and HC samples were also run alongside to monitor intra- and inter-experimental variations.

### MALDI-TOF mass spectrometry analysis

Sample measurements were performed in an Autoflex III MALDI-TOF/TOF mass spectrometer (Bruker Daltonics, Leipzig, Germany) equipped with a 200-Hz Smart beam laser and using the Flex control v.3.4 software. Samples were analyzed with manual laser positioning. Spectra were generated by averaging 1000 single laser shots (100 shots at 10 different spot positions) at a laser frequency of 200 Hz and detected in linear positive mode. The IS1 voltage was 20 kV, the IS2 voltage was maintained at 18.4 kV, the lens voltage was 6.5 kV, and the extraction delay time was 180 ns. Protein peaks between 2–10 kDa were selected for analysis. Mass accuracy was calibrated externally using the Protein Calibration Standard I and the Bacterial Test Standard, from Bruker (Madrid, Spain).

### Data pre-processing and feature selection

Pre-processing of raw mass spectra, peak detection and alignment were performed using *MALDIquantForeign* and *MALDIquant* packages [29] in R [30]. Processing of single spectrum included the square root transformation for variance stabilization, a Savitzky-Golay filter to smooth the spectra, the SNIP algorithm to correct the baseline, and the normalization of the intensity values by the Total-Ion-Current (TIC) calibration. In the spectral alignment step, a series of spectral peaks appearing with a frequency greater than 90% in the training set was used as a reference. The criteria applied to align, detect and bin peaks were 0.17% tolerance in mass accuracy and a signal-to-noise ratio of 3. After spectra alignment and binning, peaks with a frequency greater than 50% were selected as spectral features, and the corresponding intensity matrix used for further statistical analysis.

### Quality control of spectra

A Pearson correlation matrix was used for intra-experimental quality control of technical sampling replicates [31]. For every MGUS and HC serum sample, an average correlation coefficient of the technical replicates (*r*_*avg*_) was computed. A correlation threshold for high quality spectra was defined as *r*_*th*_ *= μ-3σ*, where *μ* is the mean average correlation coefficient of all serum samples and *σ* is the standard deviation. Serum samples containing low quality spectra had an average correlation coefficient lower than the correlation threshold (*r*_*avg*_*<r*_*th*_). Technical sampling replicates that poorly correlate with the rest were removed. Single serum samples with just one technical replicate passing the quality control were discarded, and biological replicates were mandatory for the analysis.

The inter-experimental quality control was evaluated with a set of blood serum samples analyzed by MALDI-TOF on different dates. For each MGUS and HC biological replicate, an average correlation coefficient of its technical replicates was computed. The mean average correlation coefficient of technical replicates of inter-experimental serum samples was used as a measure of reproducibility of the MALDI-TOF analysis.

### Predictive model building and assessment

Pre-processed spectra that passed the quality control formed a labeled set of protein profiles. A matrix (peak intensities versus featured m/z) was created and all data were used to develop a predictive model for serum sample classification into MGUS or HC predicted classes. The predictive model building was implemented by using the *e1071* and *caret* R packages [32,33].

A Support Vector Machine (SVM) model was built using a third order polynomial kernel. Three parameters were tuned, two related to the kernel definition (*gamma* and *coef0*) and one (*cost*) that sets the error penalty of the model in the optimization procedure. Technical replicates of every MGUS and HC serum sample were used as independent input vectors for the predictive model. To classify a serum sample, the predictive model first classified each of its technical replicates and then a majority-voting scheme was applied to assign the predicted serum sample class (MGUS or HC).

**Fig 1:**
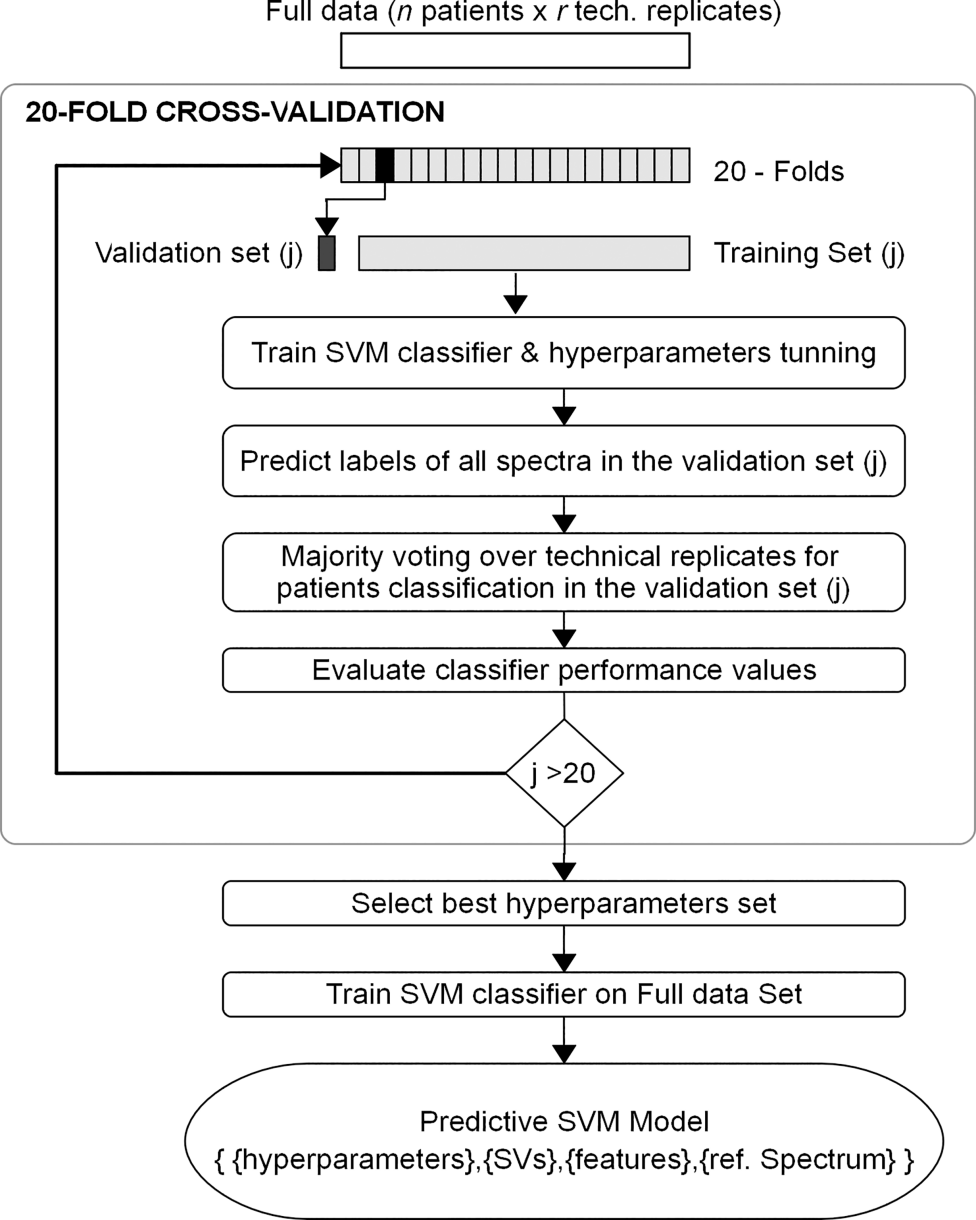
20-fold cross-validation scheme.

A 20-fold cross-validation scheme (Fig 1) was used for model performance assessment, statistical validation and model parameters tuning [34–36]. For this purpose, the full data set of technical replicates of all serum samples was randomly split into 20 completely separate folds. Technical replicates of each biological sample were restricted to the same fold to prevent over-fitting of the classifier. All folds except one–that was held out to act as validation set–were used to train and tune the SVM model that was then applied to predict the excluded validation set. The procedure was repeated 20 times, treating each time a different fold as validation set. Numerical performance measures were estimated each time. The parameters associated with the best performance estimates were chosen to train the SVM classifier on the full data set. The resulting predictive model was defined as the trained polynomial kernel SVM complemented with the spectral features set and the reference peaks.

The classifier performance measures were based on the confusion matrix method that cross-tabulates the biological group and predicted class for the serum samples, considering MGUS and HC as positive and negative class (Tables 2 and 3).

**Table 2:** Confusion matrix.

		Biological group	
		MGUS	HC
**Predicted class**	MGUS	True Positive (TP)	False Positive (FP)
	HC	False Negative (FN)	True Negative (TN)

Each cell represents a count of predictions falling into the corresponding category (MGUS or HC).

**Table 3:** Classifier performance measures based on the confusion matrix method.

Accuracy	$\frac{TP+TN}{TP+TN+FP+FN}$
Sensitivity (True Positive Rate)	$\frac{TP}{TP+FN}$
Specificity (True Negative Rate)	$\frac{TN}{TN+FP}$

Accuracy was computed as the proportion of correctly classified samples. Sensitivity and specificity were computed as the rate of correctly predicted samples in the positive and negative labeled class, respectively (TP: true positive; TN: true negative; FP: false positive; FN: false negative).

**Fig 2:**
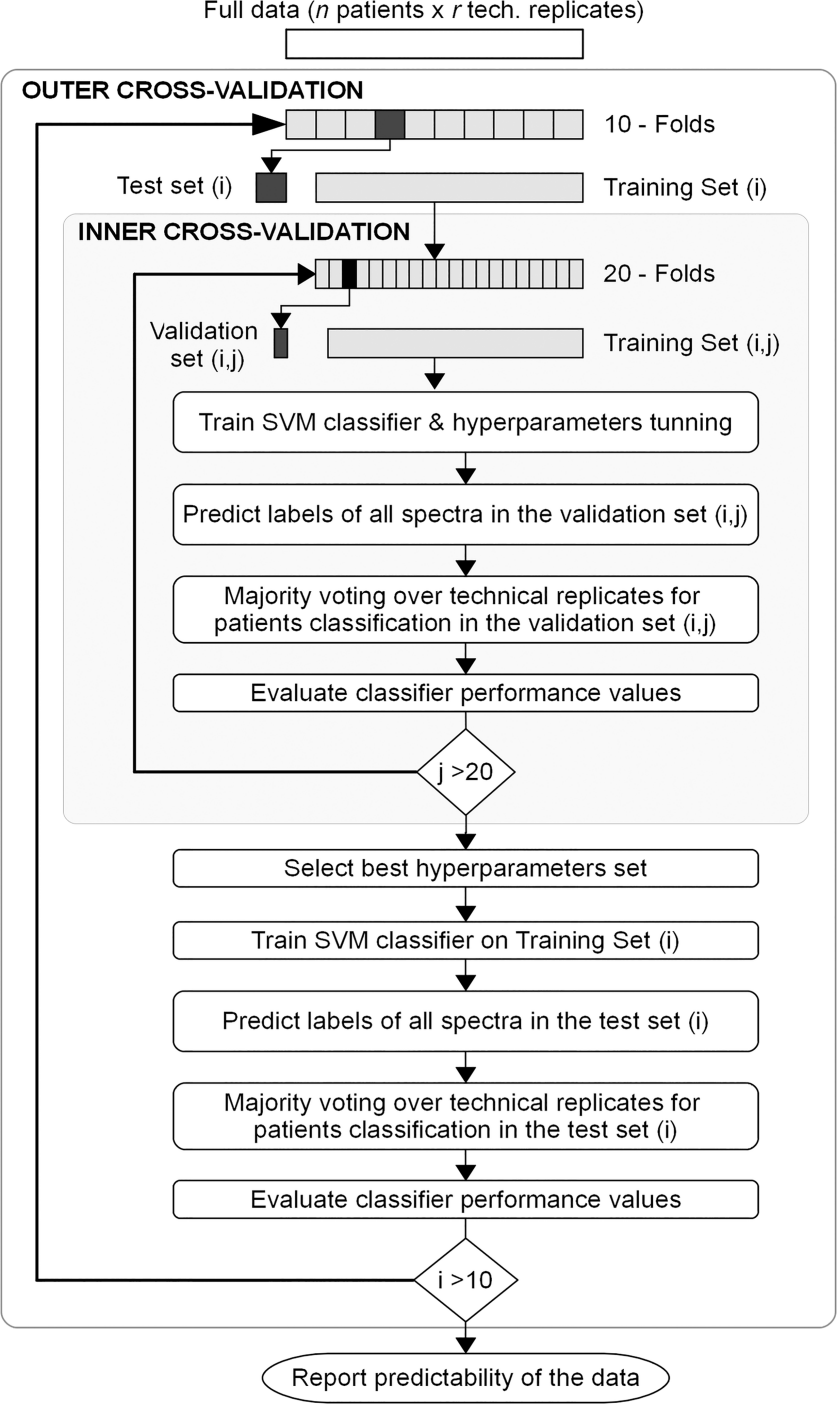
Double cross-validation scheme. It highlights the two nested loops. The outer cross-validation loop provides 10 performance estimates from predicting the corresponding test set by the optimized model built in the inner 20 fold cross-validation loop. The data set used to build and tune the model in the inner cross-validation loop is completely independent of the test set used in the outer iteration.

The classification of an independent blinded test set validated the generalization ability of the predictive model. The generalization performance and the data predictability (statistical dependence between protein profiles and class labels) were estimated by a double cross-validation method [34,37]. Fig 2 shows the two nested loops scheme. In the outer cross-validation loop, the full data set was randomly split into 10 completely separate folds considering the technical replicates restriction indicated above. One fold was held out to act as an independent test set for each iteration. The remaining nine folds were used as training set to perform the 20-fold inner cross-validation loop to optimize the model parameters. Once the best parameters for the iteration were selected, the resulting SVM classifier was trained on the current training set and applied to classify the corresponding test set. As a result, 10 performance estimates were obtained from the outer cross-validation loop. In each outer iteration, test samples were completely independent of the training set used in the inner cross-validation loop.

## Results

### Feature selection and quality control

Raw MALDI-TOF spectra from the 95 MGUS and 100 HC serum samples in the training set were pre-processed as described in Materials and Methods. A total of 765 mass spectra from technical replicates were analyzed. A set of 13 m/z peaks appearing with a frequency greater than 90% were selected as reference peaks to align serum samples spectra (Table 4A). After alignment and binning, a set of 40 m/z peaks appearing with a frequency greater than 50% were chosen as spectral features (Table 4B). Then, a feature matrix was generated which contained the intensities corresponding to the featured m/z spectral values for every technical replicate. The matrix, with all rows labeled with the serum sample identification and the biological group flag (MGUS or HC), was used for further statistical analysis and to build the predictive model.

**Table 4:** Spectral peaks selected in the analysis of technical replicates mass spectra.

**A**	m/z (Da)									
	2554.40	2660.76	2755.04	3192.90	3242.07	3263.82	3954.33	4092.44	4211.37	5906.25
	6434.47	6632.72	7767.02							
**B**	m/z (Da)									
	2021.69	2082.35	2114.80	2192.65	2209.83	2378.88	2495.96	2554.40	2604.38	2641.45
	2660.76	2723.83	2755.04	2769.70	2863.08	2884.92	2933.20	2954.46	3159.17	3192.90
	3215.50	3242.07	3263.82	3449.01	3884.10	3954.33	4055.61	4092.44	4211.37	4269.32
	4283.17	4644.92	4965.40	5338.46	5906.25	6434.47	6632.72	7767.10	9133.84	9290.47

(A) Set of m/z reference peaks with a frequency greater than 90% used for spectra alignment.

(B) Set of m/z spectral features with a frequency greater than 50% used to build the feature matrix for statistical analysis.

A quality control procedure was applied to detect and discard technical sampling replicates with low quality spectra in the pre-processed training set. For intra-experimental serum samples spectra, we obtained a mean average correlation coefficient *μ =* 0.984 and a standard deviation *σ* = 0.028. Then a correlation threshold (*r*_*th*_ *= μ-3σ*) of 0.9 was established. Serum samples with an average correlation coefficient of technical replicates *r*_*avg*_*<r*_*th*_ indicated that some technical replicates correlated poorly with the rest, so they were removed (2.3% and 1.3% of MGUS and HC technical replicate spectra). No single serum sample was discarded. Additionally, an inter-experimental quality control of biological serum samples was performed on a set of 12 MGUS and 11 HC sera. A mean average correlation coefficient of 0.934 was obtained. The strong correlation obtained for the inter-experimental set validated the reproducibility of MALDI-TOF analysis.

### Predictive model

A total of 751 technical replicate spectra passed the quality control, generating a labeled set of serum protein profiles used to build the predictive model for classification of serum samples into MGUS and HC classes. Our model was based on support vector machine (SVM) and tuned applying a 20-fold cross-validation scheme (Fig 1). Table 5 shows the 20 performance estimates of the classifier each one corresponding to an iteration of the cross-validation scheme (see also Table 3). The parameters resulting in the best performance (shaded in Table 5) were selected to train the final SVM predictive model on the full data set.

**Table 5:** Classifier performance estimates obtained from the 20-fold cross-validation scheme.

Fold #	gamma	coef0	cost	Sensitivity	Specificity	Accuracy	p-value
**1**	0.00010	0.12	150	1.00	1.00	1.00	0.0077
**2**	0.00060	0.09	150	1.00	0.86	0.93	0.0009
**3**	0.00005	0.13	175	1.00	0.50	0.67	0.6503
**4**	0.00005	0.09	175	1.00	0.75	0.88	0.0021
**5**	0.00005	0.15	185	1.00	0.80	0.92	0.0166
**6**	0.00005	0.12	160	1.00	0.67	0.83	0.1094
**7**	0.00005	0.09	160	1.00	0.40	0.67	0.3743
**8**	0.00005	0.09	185	1.00	0.44	0.58	0.9456
**9**	0.00005	0.08	190	0.70	0.80	0.73	0.4041
**10**	0.00005	0.12	180	1.00	0.86	0.94	0.0016
**11**	0.00005	0.13	180	1.00	0.50	0.63	0.8862
**12**	0.00005	0.40	240	1.00	1.00	1.00	0.0016
**13**	0.00005	0.15	120	0.83	1.00	0.88	0.3671
**14**	0.00005	0.20	170	1.00	0.60	0.80	0.0547
**15**	0.00020	0.90	240	1.00	0.86	0.92	0.0039
**16**	0.00005	0.40	120	1.00	1.00	1.00	0.0050
**17**	0.00005	0.20	90	1.00	0.80	0.89	0.0413
**18**	0.00030	1.10	240	1.00	0.83	0.93	0.0046
**19**	0.00050	0.90	200	1.00	1.00	1.00	0.0199
**20**	0.00005	0.30	120	1.00	0.83	0.92	0.0039

The tuned parameters (gamma, coef0, cost) and the performance estimates (sensitivity, specificity, accuracy) for each iteration are shown. The parameters corresponding to the best performance are shaded. A p-value from McNemar's Chi-square test was computed, and p < 0.05 was considered statistically significant.

We used the resulting predictive model to classify a blinded test set consisting of 8 MGUS and 8 HC randomly selected samples. Technical replicates were previously pre-processed and the quality control procedure applied. Table 6 shows the biological group and the predicted class label for the samples in the blinded test set. Predicted class for each serum sample was the result of majority voting applied to the labels assigned to its technical replicates. The predictive model used to classify blinded test samples showed 88% accuracy, 75% sensitivity and 100% specificity.

**Table 6:** Biological group and predicted class label for serum samples in the blinded test set.

**Serum sample**	1T	2T	3T	4T	5T	6T	7T	8T	9T	10T	11T	12T	13T	14T	15T	16T
**Biological group**	HC	HC	HC	MGUS	MGUS	MGUS	MGUS	HC	HC	HC	MGUS	MGUS	MGUS	MGUS	HC	HC
**Predicted class**	HC	HC	HC	MGUS	MGUS	HC	HC	HC	HC	HC	MGUS	MGUS	MGUS	MGUS	HC	HC

Blinded test samples were identified as *nT* to mask any information about the biological group before their classification. False negative results are shaded.

The limited size of the blinded test set prevented us from obtaining a reliable estimate of the model generalization performance and of the dependence between MALDI-TOF protein profiles and class labels. Consequently, we used a double cross-validation method to overcome such limitation (Fig 2). Table 7 shows the 10 performance estimates obtained from the outer cross-validation loop. Those estimates were obtained by predicting a test set completely independent of the data set used to build and tune the model in the inner cross-validation loop. The average model accuracy was 88% and the average sensitivity and specificity were 89% and 86%, respectively.

**Table 7:** Performance estimates obtained from the double cross-validation method.

Outer fold #	Sensitivity	Specificity	Accuracy	p-value
**1**	1.00	1.00	1.00	0.0000001
**2**	0.64	0.93	0.80	0.0111700
**3**	1.00	0.86	0.94	0.0013510
**4**	1.00	0.75	0.89	0.0002533
**5**	0.82	1.00	0.92	0.0001199
**6**	0.91	0.82	0.86	0.0004277
**7**	0.62	0.69	0.65	0.0843200
**8**	1.00	0.90	0.92	0.1618000
**9**	1.00	0.89	0.95	0.0001114
**10**	0.94	0.80	0.88	0.0025660
**Average**	0.89	0.86	0.88	
**Std. Dev**	0.15	0.10	0.10	

The performance estimates (sensitivity, specificity, accuracy) for every outer iteration and the corresponding average values are shown. A p-value from McNemar's Chi-square test was computed and p < 0.05 was considered statistically significant.

**Fig 3:**
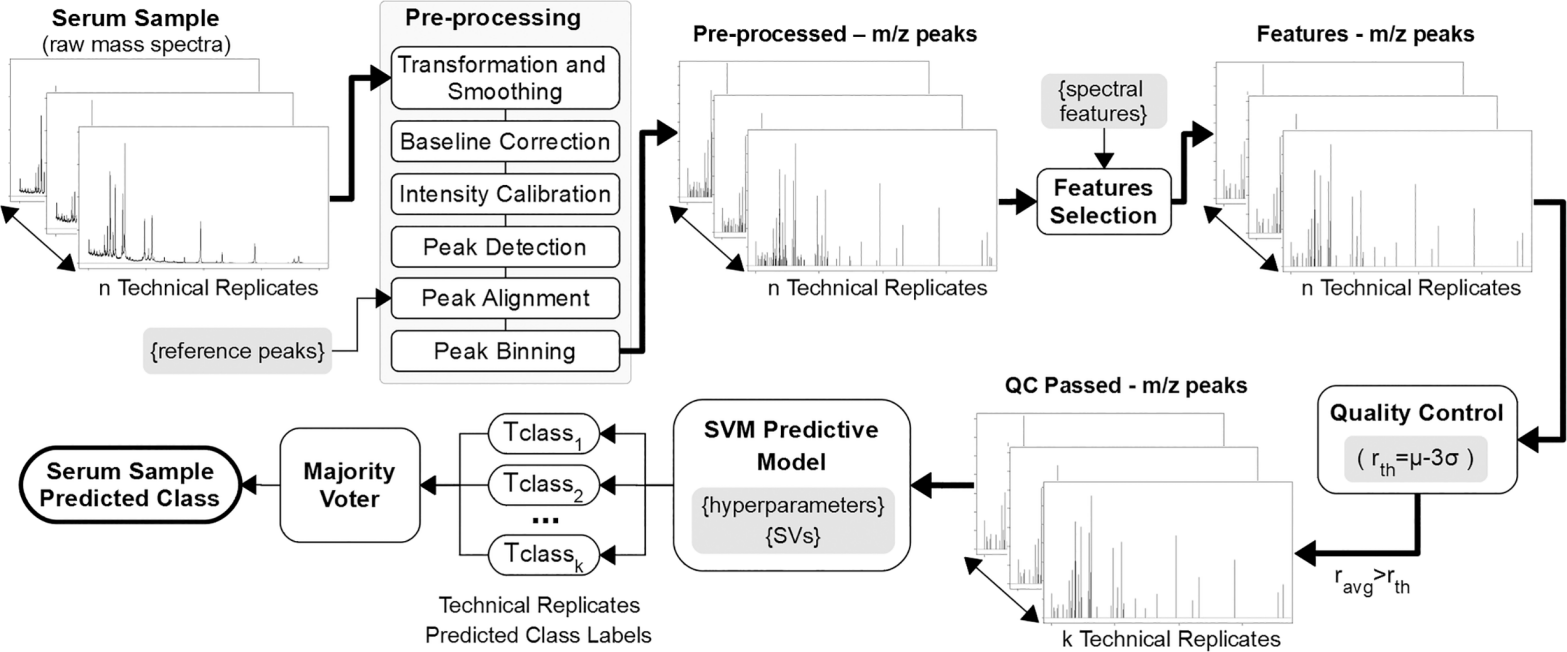
Classification process of an unknown serum sample. *n* technical replicates from serum sample raw mass spectra are pre-processed and features selected. The correlation threshold (*r*_*th*_) sets the *k* technical replicates passing the intra-experimental quality control (QC). The SVM predictive model classifies the *k* technical replicates. The majority voter assigns the serum sample predicted class. The parameters determined from the processing of the training set and the building of the predictive model are shaded.

The process used to classify an unknown serum sample into MGUS or HC class is shown in Fig 3. The *n* technical replicates spectra are pre-processed, using the set of m/z reference peaks (Table 4A) for peak alignment. Then the features for every technical replicate are selected, corresponding to the featured m/z spectral values (Table 4B). Next the quality control is applied to discard technical replicates with low quality spectra. The *k* technical replicates passing the quality control are classified by the developed predictive model and finally a majority-voting scheme assigns the predicted serum sample class.

In summary, our predictive model which was built using an SVM machine learning method, tuned by a cross-validation scheme and double cross-validated, allowed us to classify serum samples as belonging to either MGUS or HC individuals. Predicting a blinded test set validated our model, which capacity for classifying new serum samples was proved by the average performance results from the double-cross validation scheme.

## Discussion

The composition of the serum peptidome can reflect what occurs in cellular and organ systems [38]. Direct profiling of serum peptides by MALDI-TOF can be used to identify biomarkers of disease and to classify samples into disease and treated groups [23,39]. We have analyzed the serum peptidome profile of both MGUS patients and healthy control (HC) volunteers by MALDI-TOF mass spectrometry. The biological variability of MGUS and HC serum samples yields highly complex information in the mass spectra, thus making difficult to discriminate between both groups and to identify proteins suitable as putative biomarkers of MGUS. Therefore, we present a method based on a machine learning technique to analyze mass spectrometry-derived proteomic data and to classify serum samples into MGUS or HC classes. We aimed to achieve the highest prediction quality without analyzing the biological significance of the serum spectral features. The predictive model was developed to classify unknown serum samples into MGUS or HC classes, based on Support Vector Machine (SVM), a supervised machine learning method widely used to classify samples in clinical proteomics [34,40].

As a first step in the sample analysis protocol, we have applied a quality control to detect and discard low-quality spectra before any statistical analysis and model building. Because protein expression profiles obtained from technical replicates should be similar, we have used the Pearson correlation matrix of intra-experimental spectra as a quality control tool to differentiate good and poor spectra. The results indicated a low ratio of discarded technically replicated spectra in both MGUS and HC groups (2.3% and 1.3%). Reproducibility of the MALDI-TOF analyses was validated by the computed inter-experimental mean average correlation coefficient, with a strong correlation (0.934) observed among the biological replicates of the serum samples.

We used the pre-processed spectra from the training set that passed the quality control to develop the SVM predictive model. A 20-fold cross-validation re-sampling method was used to train and test the predictive model to make an optimal use of the spectral data (Fig 1). Our model first classified technical replicates of the mass spectra and afterwards a majority-voting scheme assigned the predicted serum sample class, which provided robustness to the classification procedure. To validate the generalization ability of the predictive model, a blinded test set, not used in the model building, was classified (Table 6). We have applied a double cross-validation scheme (Fig 2) to overcome the shortcomings arising from the limited size of the blinded test set. This resulted in 10 performance estimates, each one predicting test samples independent of the model building (Table 7). The double cross-validated average model accuracy (88%) and the average sensitivity and specificity (89% and 86%) confirmed the statistical dependence between MALDI-TOF peptidome profiles of MGUS and HC serum samples and class labels. We have demonstrated the generalization ability of the predictive model to classify unknown serum samples. Therefore, our model can be used as a suitable classifier for predicting MGUS dyscrasia in any serum sample. Our results provide further evidence that MALDI-TOF mass spectrometry can be used to distinguish MGUS in serum samples [12].

We showed elsewhere that MGUS can be detected and characterized using differential scanning calorimetry (DSC) [13], a technique that can also characterize the progress of MGUS patients to related pathologies [13,17,18]. In fact, using DSC and mass spectrometry together is grasped as a potent tool for detecting a variety of pathologies in human blood samples [41]. Our study, based on MALDI-TOF analysis and a machine learning predictive model, provides further support for using mass spectrometry to classify unidentified serum samples, which can be applied to the clinical diagnosis of MGUS.
